# Fullerene modification CdSe/TiO_2_ and modification of photocatalytic activity under visible light

**DOI:** 10.1186/1556-276X-8-189

**Published:** 2013-04-23

**Authors:** Ze-Da Meng, Lei Zhu, Shu Ye, Qian Sun, Kefayat Ullah, Kwang-Youn Cho, Won-Chun Oh

**Affiliations:** 1 Department of Advanced Materials Science and Engineering, Hanseo University, Seosan, Chungchungnam, 356-706, South Korea; 2 Korea Institute of Ceramic Engineering and Technology, Seoul, 153-801, South Korea

**Keywords:** Fullerene, CdSe, Visible light, UV–vis, TEM

## Abstract

CdSe, CdSe-TiO_2_, and CdSe-C_60_/TiO_2_ composites were prepared using sol–gel method, and their photocatalytic activity was evaluated by measuring the degradation of rhodamine B solutions under visible light. The surface area, surface structure, crystal phase, and elemental identification of these composites were characterized by nitrogen adsorption isotherms, scanning electron microscopy (SEM), transmission electron microscopy (TEM), X-ray diffraction (XRD), energy dispersive X-ray spectroscopy (EDX), and UV-visible (vis) absorption spectrophotometry. XRD showed that the CdSe-C_60_/TiO_2_ composite contained a typical single and clear anatase phase. SEM of the CdSe-C_60_/TiO_2_ composites revealed a homogenous composition in the particles. EDX revealed the presence of C and Ti with strong Cd and Se peaks in the CdSe-C_60_/TiO_2_ composite. The degradation of dye was determined by UV–vis spectrophotometry. An increase in photocatalytic activity was observed and attributed to an increase in the photoabsorption effect by fullerene and the cooperative effect of the CdSe. The repeatability of photocatalytic activity was also tested in order to investigate the stability of C_60_ and CdS-C_60_/TiO_2_ composites.

## Background

TiO_2_ is the most widely used photocatalyst for effective decomposition of organic compounds in air and water under irradiation of UV light with a shorter wavelength, corresponding to its bandgap energy, due to its relatively high photocatalytic activity, biological and chemical stability, low cost, nontoxic nature, and long-term stability. However, the photocatalytic activity of TiO_2_ (the bandgap of anatase TiO_2_ is 3.2 eV which can be excited by photons with wavelengths below 387 nm) is limited to irradiation wavelengths in the UV region [1,2]. However, only about 3% to 5% of the solar spectrum falls in this UV range. This limits the efficient utilization of solar energy for TiO_2_. Some problems still remain to be solved in its application, such as the fast recombination of photogenerated electron–hole pairs. Therefore, improving the photocatalytic activity by modification has become a hot topic among researchers in recent years [3,4].

Photosensitization of stable, large bandgap semiconductors such as SnO_2_, TiO_2_, and ZnO in visible light using semiconducting photosensitizers such as CdS, CdSe, and CdTe [5] has been a long-sought, continuing goal in the area of photoelectrochemical solar energy conversion. Cadmium selenide is a kind of semiconductor with a forbidden zone of 1.7 eV, and its valence electrons can be easily evoked to conduction band when the light wavelength of evoking light is ≤730 nm [6-9]. However, in practical applications, the photoelectrical properties and photocatalytic efficiency of CdSe require improvement.

Conjugated material is proposed to be a good candidate for improving the transportation of photocarriers in the photocatalysis process by forming an electronic interaction with TiO_2_ due to its unique properties in electron or hole transporting [10]. Among them, fullerene has a variety of special chemical and physical properties due to its delocalized conjugated structure and has been studied quite extensively [11]. Fullerene can efficiently promote a rapid photo-induced charge separation and slow down charge recombination in the dark. Therefore, fullerene has been used to raise the performances of solar cell and medicinal chemistry [12,13]. Kamat et al. have demonstrated the charge transfer between fullerene clusters and titanium dioxide under visible light; fullerene can be reduced by one-electron function in colloidal TiO_2_ suspensions and form C_60_[14]. Besides, many works focused on improving the efficiency of dye sensitization-based photochemical solar cells by adding C_60_. Those researches were mostly focused on the electron transfer between TiO_2_ particle and C_60_ cluster. Photon conversion efficiency can be improved by C_60_ cluster due to high separation efficiency for the photo-induced electrons and holes. Although the use of fullerene for scavenging photogenerated electrons from titanium dioxide particles has been demonstrated, a few efforts are made to utilize the unique properties of fullerenes to increase the efficiency of photocatalysis; however, the interior mechanism is yet not very clear. A systematical study on a purpose of understanding the interaction between C_60_ molecules and TiO_2_ and further effect on the photocatalytic activity is still necessary and important [15-17].

In this work, CdSe-TiO_2_ and C_60_-hybridized CdSe-TiO_2_ photocatalysts showed significantly enhanced photocatalytic activity for the degradation of salicylic acid and formaldehyde under visible-light irradiation. The enhancement of photoactivity was attributed the photosensitization of CdSe and the enhanced interfacial charge separation between C_60_ layers and TiO_2_ particles.

## Experimental

### Materials

Crystalline fullerene (C_60_) powder of 99.9% purity from TCI (Tokyo Kasei Kogyo Co. Ltd., Tokyo, Japan) was used as the carbon matrix. For the oxidization of C_60_, *m*-chloroperbenzoic acid (MCPBA) was chosen as the oxidizing agent and was purchased from Acros Organics (Fair Lawn, NJ, USA). Benzene (99.5%) was used as the organic solvent and was purchased from Samchun Pure Chemical Co., Ltd. (Seoul, Korea). Cadmium acetate dihydrate (Cd(CH_3_COO)_2_, 98%), selenium metal powder, and ammonium hydroxide (NH_4_OH, 28%) were purchased from Dae Jung Chemicals & Metal Co., Ltd. (Siheung-si, Gyonggi-do, Korea). Anhydrous purified sodium sulfite (Na_2_SO_3_, 95%) was purchased from Duksan Pharmaceutical Co., Ltd. (Ansan-si, Gyeonggi-do, Korea). Titanium(IV) n-butoxide (TNB, C_16_H_36_O_4_Ti) as the titanium source for the preparation of the CdSe-C_60_/TiO_2_ composites was purchased as reagent-grade from Acros Organics (USA). Rhodamine B (Rh.B, C_28_H_31_ClN_2_O_3_) was purchased from Samchun Pure Chemical Co., Ltd. (Korea). All chemicals were used without further purification, and all experiments were carried out using distilled water.

### Synthesis of CdSe

For the synthesis of CdSe, sodium selenosulfite (Na_2_SeSO_3_) solution and Cd(NH_3_)_4_^2+^ solution were first prepared. Na_2_SO_3_ (4 g) and selenium metal powder (0.2 g) were dissolved in 20 of mL distilled water and refluxed for 1 h to form Na_2_SeSO_3_ solution. Meanwhile, Cd(CH_3_COO)_2_ (0.675 g) was dissolved in 7 mL of distilled water. NH_4_OH (2 mL) was added, and the mixture was stirred until it dissolved completely to form Cd(NH_3_)_4_^2+^ solution. Finally, the Cd(NH_3_)_4_^2+^ and Na_2_SeSO_3_ solutions were mixed together, and the mixture was stirred and refluxed for at least 5 h. After the mixture had been brought down to room temperature, the mixture was filtered through a Whatman filter paper. The solids obtained were collected and washed five times with distilled water. After being dried in vacuum at 353 K for 8 h, the CdSe compound was obtained.

### Synthesis of CdSe-C_60_ composite

For the preparation of the CdSe-C_60_ composite, C_60_ had to be functionalized by MCPBA at first. MCPBA (*ca*. 1 g) was suspended in 50 mL of benzene, followed by the addition of fullerene (*ca*. 30 mg). The mixture was heated under reflux in air and stirred for 6 h. The solvent was then dried at the boiling point of benzene (353.13 K). After completion, the dark-brown precipitates were washed with ethyl alcohol and dried at 323 K, resulting in the formation of oxidized fullerene.

**Figure 1:**
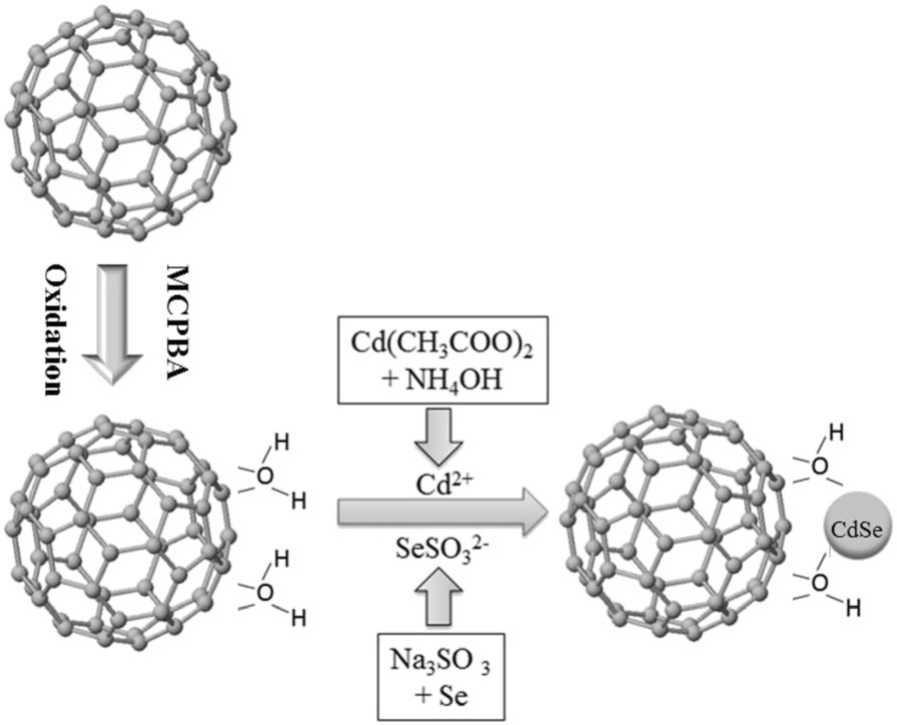
Schematic presentation of the functionalization of fullerenes and the coupling of CdSe nanoparticles with fullerenes.

The functionalized C_60_ with the Cd(NH_3_)_4_^2+^ and Na_2_SeSO_3_ solutions prepared as previously described were mixed together, and the mixture was stirred and refluxed for at least 5 h. After the mixture had been brought down to room temperature, the mixture was filtered through a Whatman filter paper. The solids obtained were collected and washed five times with distilled water. After being dried in a vacuum at 353 K for 8 h, a CdSe-C_60_ composite with chemical band was obtained. Figure 1 shows the schematic presentation of the functionalization of C_60_ and the coupling of CdSe nanoparticles with C_60_. Figure 1 shows the schematic presentation of the functionalization of MWCNTs and the coupling of CdSe nanoparticles with MWCNTs.

### Synthesis of CdSe-C_60_/TiO_2_ composites

CdSe-C_60_ was prepared using pristine concentrations of TNB for the preparation of CdSe-C_60_/TiO_2_ composites. CdSe-C_60_ powder was mixed with 3 mL TNB. The solutions were homogenized under reflux at 343 K for 5 h while being stirred in a vial. After stirring, the solution transformed to CdSe-C_60_/TiO_2_ gels and was heat-treated at 873 K to produce the CdSe-C_60_/TiO_2_ composites.

### Characterization

X-ray diffraction (XRD; Shimadzu XD-D1, Uki, Kumamoto, Japan) was used to identify the crystallinity of the composite with monochromatic high-intensity Cu Ka radiation (*l* = 1.5406 Å). Scanning electron microscopy (SEM; JSM-5600, JEOL Ltd., Tokyo, Japan) was used to observe the surface state and structure of the prepared composite using an electron microscope. Transmission electron microscopy (TEM; JEM-2010, JEOL Ltd.) was used to determine the state and particle size of the prepared composite. TEM at an acceleration voltage of 200 kV was used to investigate the number and the stacking state of graphene layers on the various samples. TEM specimens were prepared by placing a few drops of sample solution on a carbon grid. The elemental mapping over the desired region of the prepared composite was determined by an energy dispersive X-ray spectroscopy (EDX) analyzer attached to the SEM. UV-visible (vis) diffuse reflectance spectra were obtained using a UV–vis spectrophotometer (Neosys-2000, Scinco Co. Ltd., Seoul, Korea) using BaSO_4_ as a reference at room temperature and were converted from reflection to absorbance spectra by the Kubelka-Munk method.

### Photocatalytic degradation of dyes

Photocatalytic activity was evaluated by dye degradation in aqueous media under visible-light irradiation. For visible-light irradiation, the reaction beaker was located axially and held in a visible lamp box (8 W, halogen lamp, KLD-08 L/P/N, Korea). The luminous efficacy of the lamp was 80 lm/W, and the wavelength was 400 to 790 nm. The lamp was located at a distance of 100 mm from the aqueous solution in a dark box. The initial concentration of the dyes was set at 1 × 10^−5^ mol/L in all experiments. The amount of photocatalytic composite used was 0.05 g/50-mL solution. The reactor was placed for 2 h in the dark box to make the photocatalytic composite particles adsorb as many dye molecules as possible. After the adsorption phase, visible-light irradiation was restarted to make the degradation reaction proceed. To perform dye degradation, a glass reactor (diameter = 4 cm, height = 6 cm) was used, and the reactor was placed on the magnetic churn dasher. The suspension was then irradiated with visible light for a set irradiation time. Visible-light irradiation of the reactor was performed for 120 min. Samples were withdrawn regularly from the reactor, and dispersed powders were removed in a centrifuge. The clean transparent solution was analyzed by a UV–vis spectrophotometer (Optizen POP, Mecasys Co., Ltd., Daejeon, Korea). The dye concentration in the solution was determined as a function of the irradiation time.

## Results and discussion

**Figure 2:**
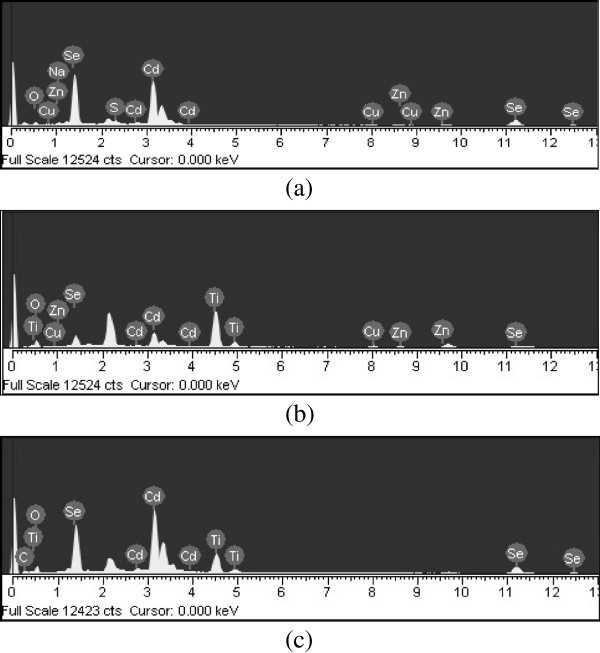
**EDX elemental microanalysis of CdSe (a), CdSe-TiO**_**2 **_**(b), and CdSe-C**_**60**_**/TiO**_**2 **_**(c), they are kinds of elements such as Cd, Se, Ti, O and C.**

The result is agreement with XRD results for titanium and CdSe. After the examinations of wounds conducted by the coated implements with SEM/EDX, special particles were found; they are kinds of elements such as Cd, Se, Ti, O and C. Table 1 lists the numerical results of EDX quantitative microanalysis of the samples. Figure 2 shows that strong Kα and Kβ peaks from the Ti element appear at 4.51 and 4.92 keV, respectively, whereas a moderate Kα peak for O was observed at 0.52 keV [18]. There were some small impurities, which were attributed to the use of fullerene without purification.

**Table 1:** EDX elemental microanalysis and BET surface area values

**Sample name**	**C (%)**	**O (%)**	**Cd (%)**	**Se (%)**	**Ti (%)**	**Impurity**	**BET (m**^**2**^**/g)**
C_60_	99.99	-	-	-	-	0.01	85.05
CdSe	-	3.41	57.37	36.45	-	2.77	26.71
CdSe-TiO_2_	-	23.57	24.34	14.52	35.46	2.14	30.47
CdSe-C_60_/TiO_2_	5.14	19.63	34.78	16.71	22.21	1.53	47.27

**Figure 3:**
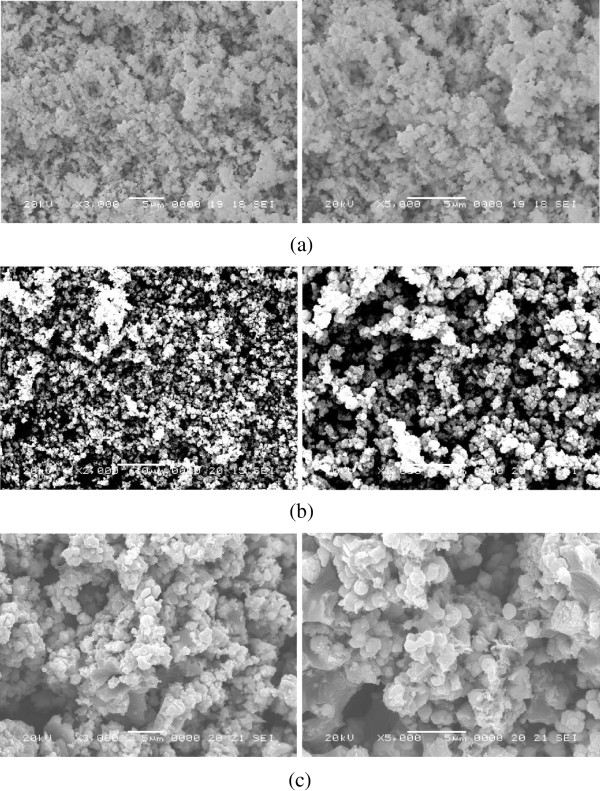
**SEM images of CdSe (a), CdSe-TiO**_**2 **_**(b), and CdSe-C**_**60**_**/TiO**_**2 **_**(c), different samples with different magnification.**

Figure 3 shows the characterized results of the microsurface structures and morphology of the CdSe, CdSe-TiO_2_, and C_60_ modified CdSe-TiO_2_ compounds. As shown in Figure 3, C_60_ and CdSe are coated uniformly on the TiO_2_ surface, which leads to an increase in nanoparticle size. Zhang et al. reported that a good dispersion of small particles could provide more reactive sites for the reactants than aggregated particles [19]. The surface roughness appears to be more with little grain aggregation. Figure 3a,b,c is CdSe, CdSe-TiO_2_, and CdSe-C_60_/TiO_2_, respectively. The aggregation phenomenon becomes increasingly serious, and the CdSe addition can make the aggregation worse. Figure 3c shows spherical C_60_ particles.

Table 1 lists Brunauer-Emmett-Teller (BET) surface areas of the raw CdSe, CdSe-TiO_2_, and CdSe-C_60_/TiO_2_ photocatalysts. The BET value decreased from 85.00 m^2^/g of pure fullerene to 47.27 m^2^/g of CdSe-C_60_/TiO_2_. The TiO_2_ and CdSe particles were introduced into the pores of fullerene, and the value of CdS-C_60_/TiO_2_ decreased [20]. Added C_60_ can increase the surface area because C_60_ has a relatively larger surface area. The BET values of CdSe and CdSe-TiO_2_ compounds were 26.71 and 30.47 m^2^/g, respectively. The BET surface area of the CdS-TiO_2_ photocatalyst was increased by 55.13 % when the CdSe-TiO_2_ particles were modified by C_60_. The pore size and pore volume increased significantly when the particles were modified by C_60_ because C_60_ particles have larger surface area and pores.

**Figure 4:**
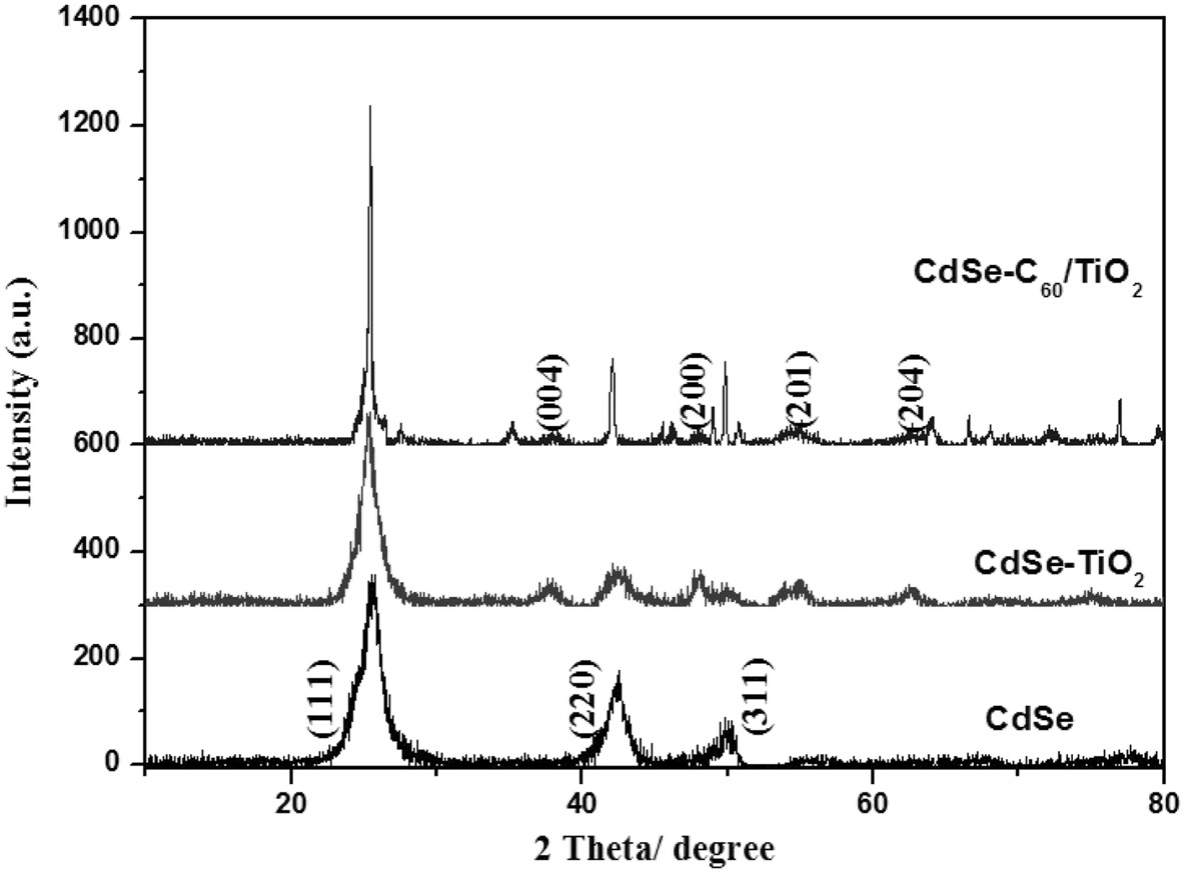
**XRD patterns of CdSe, CdSe-TiO**_**2**_**, and CdSe-C**_**60**_**/TiO**_**2**_**.**

Figure 4 shows the XRD pattern of CdSe, CdSe-TiO_2_, and C_60_-modified CdSe-TiO_2_ particles. It can be seen that the TiO_2_ modificator is of the anatase structure. It can also be seen from Figure 4 that the crystallization of the annealed TiO_2_ is worse than that of the pure TiO_2_ implanted. XRD analysis used to determine the phase purity of the samples. Figure 4 shows the XRD patterns of the component results of CdSe and CdSe-TiO_2_ photocatalysts. Figure 4 shows all of the peaks around 2*θ* of 25.4°, 42°, and 49.6°, which could be indexed to the characteristic peaks (111), (220), and (311) plane reflections of cubic crystal structure CdSe with a lattice constant of 6.05 Å (JCPDS 65–2891) [21,22]. Moreover, with the CdSe-TiO_2_ photocatalyst, some peaks were also found at 37.9°, 47.8°, 55°, and 62.7°, which could be indexed to the characteristic peaks (004), (200), (201), and (204) of anatase TiO_2_ (JCPDS 21–1272) [23,24]. No peaks for impurities were detected.

**Figure 5:**
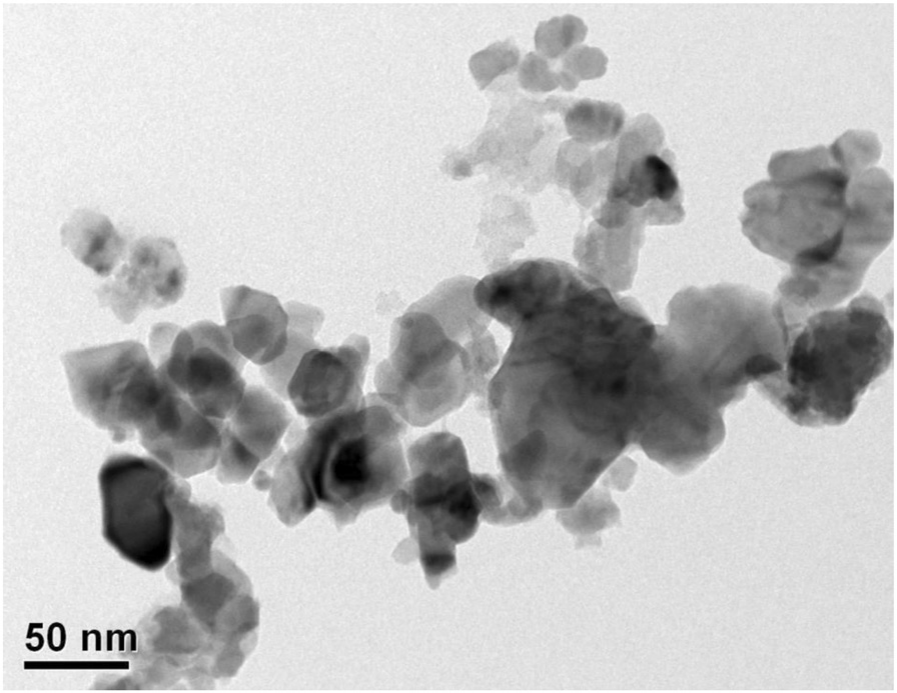
**TEM image of the CdSe-C**_**60**_**/TiO**_**2 **_**compounds.**

Figure 5 shows TEM images of CdSe-C_60_/TiO_2_. The representative TEM images in Figure 5 show that the prepared powders are uniform with some aggregations between particles. The mean diameter of C_60_ was estimated to be approximately 20 to 30 nm. From Figure 5, the image of CdSe-C_60_/TiO_2_ compounds showed that all particles had agglomerated. This suggests that the presence of CdSe and C_60_ can efficiently enhance the agglomeration of TiO_2_ and impede the dispersion of nanoparticles.

**Figure 6:**
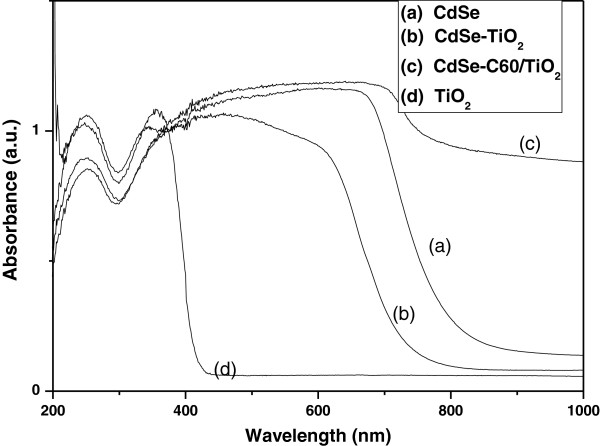
**UV–vis diffuse reflectance spectra of CdSe, CdSe-TiO**_**2**_**, TiO**_**2**_, **and CdSe-C**_**60**_**/TiO**_**2**_**.**

UV–vis reflectance analysis was carried out on various systems of interest, and the measurements were then converted to absorbance spectra using Kubelka-Munk method. Figure 6 shows the UV–vis diffuse reflectance spectra of the CdSe, CdSe-TiO_2_, TiO_2_, and CdSe-C_60_/TiO_2_. As expected, the spectrum obtained from the bare TiO_2_ shows that TiO_2_ absorbs mainly the UV light with absorption wavelength below 400 nm. After the introduction of CdSe, the absorption edge is shifted toward the visible region. The CdSe exhibits the fundamental absorption edge at about 812 nm. For CdSe-TiO_2_, the absorbance spectrum has two absorbance onsets at approximately 738 nm and 400 nm, corresponding to the presence of CdSe and TiO_2_ particles, respectively. It is interesting to note that the onset for TiO_2_ absorption was almost unchanged (at a wavelength of about 400 nm) while the CdSe absorbance onset at 812 nm was a blueshift to the wavelength of 738 nm. This indicated an increase in the bandgap of CdSe due to the introduced TiO_2_. CdSe-C_60_/TiO_2_ exhibits the good adsorption effect at visible region because of the synergistic reaction of CdSe, C_60_, and TiO_2_.

The diffuse reflectance spectra of the CdSe, CdSe-TiO_2_, TiO_2_, and CdSe-C_60_/TiO_2_ were transformed by performing the Kubelka-Munk transformation of the measured reflectance according to the following equation:

(1) $$K={\left(1-R\right)}^{2}/2R=F\left(R\right),$$

where *K* is the reflectance transformed according to the Kubelka-Munk function, *R* is the reflectance (%), and *F*(*R*) is the so-called remission or Kubelka-Munk function [25].

It is well known that the bandgap *E*_g_ and the absorption coefficient *α* are related as in the following equation:

(2) $$\mathrm{\alpha h\nu}=A{\left(\mathrm{h\nu}-E_{g}\right)}^{\frac{1}{2}},$$

where *α*, *v*, *E*_g_, and *A* are the absorption coefficient, light frequency, bandgap, and a constant, respectively. If the compound scatters in a perfectly diffuse manner, *K* becomes equal to 2*α*. In this case, we can use the following expression:

(3) $${\left[F\left(R\right)h\nu \right]}^{2}=A\left(h\nu -E_{g}\right).$$

**Figure 7:**
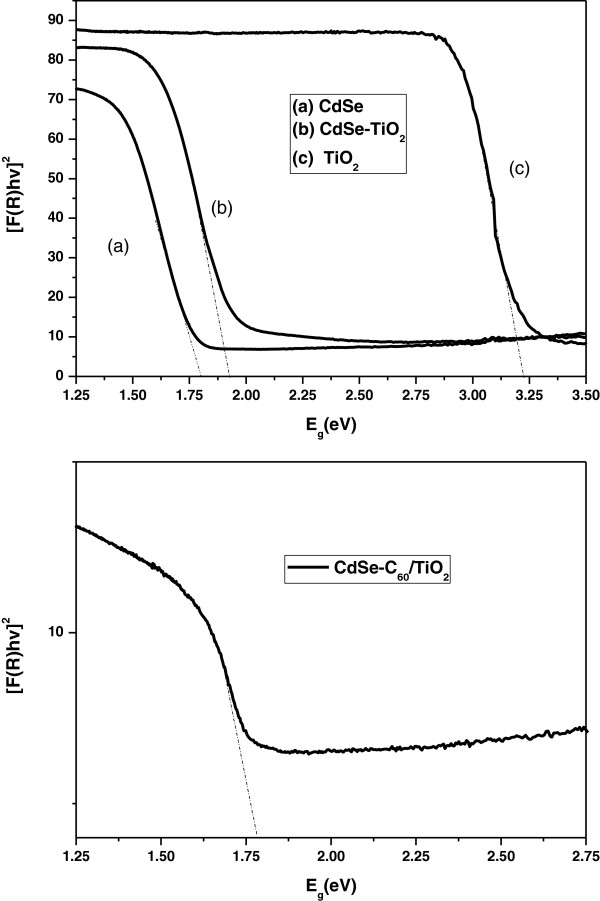
**Variation of (*α*hν)**^**2 **^**versus photon energy (hν) for CdSe, CdSe-TiO**_**2**_**, TiO**_**2**_, **and CdSe-C**_**60**_**/TiO**_**2**_**.**

Therefore, the bandgap energy (*E*_g_) of the resulting samples can be estimated from a plot of [*F*(*R*)hν]^2^ versus photon energy (hν). The [*F*(*R*)hν]^2^ versus hν graph of CdSe, CdSe-TiO_2_, TiO_2_, and CdSe-C_60_/TiO_2_ are presented in Figure 7. The intercept of the tangent to the *x*-axis would give a good approximation of the bandgap energy of the samples. The bandgap of CdSe is evaluated to be 1.81 eV, which is fairly close to the literature value of 1.74 eV [26,27]. It is also found that the bandgap of CdSe-TiO_2_ is 1.95 eV, which is greater than the standard bandgap (1.78 eV for CdSe), showing a blueshift of 0.14 eV. The bandgap of CdSe-C_60_/TiO_2_ is about 1.77 eV, showing a blueshift of 0.05 eV.

**Figure 8:**
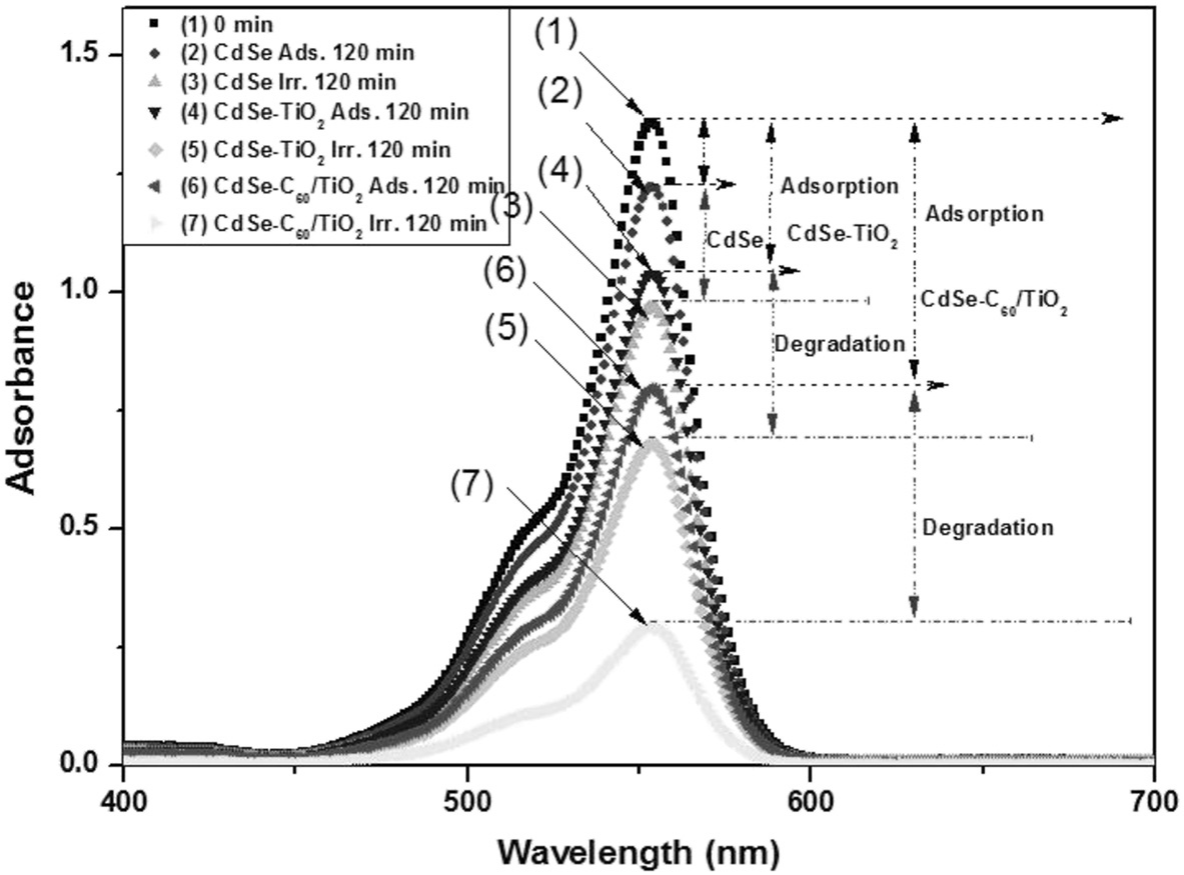
**UV–vis spectra of the Rh.B concentration against CdSe, CdSe-TiO**_**2**_, **and CdSe-C**_**60**_**/TiO**_**2 **_**composites.**

Figure 8 shows the time series of dye degradation using CdSe, CdSe-TiO_2_, and CdSe-C_60_/TiO_2_ under visible-light irradiation. The spectra for the dye solution after visible-light irradiation show the relative degradation yields at different irradiation times. The decrease in dye concentration continued with an oppositely gentle slope, which was due to visible-light irradiation. The concentration of dyes was 1.0 × 10^−5^ mol/L, and the absorbance for dye decreased with the visible-light irradiation time. Moreover, the dye solution increasingly lost its color, and the dye concentration decreased. Two steps are involved in the photocatalytic decomposition of dyes: the adsorption of dye molecules and degradation. After adsorption in the dark for 30 min, the samples reached adsorption-desorption equilibrium. In the adsorptive step, CdSe, CdSe-TiO_2_, and CdSe-C_60_/TiO_2_ composites showed different adsorptive effects with CdSe-C_60_/TiO_2_ having the best adsorptive effect. The adsorptive effect of pure CdSe was the lowest. The adsorptive effect of CdSe-C_60_/TiO_2_ was better than that of CdSe-TiO_2_ because the added C_60_ can enhance the BET surface area which can increase the adsorption effect. CdSe-C_60_/TiO_2_ has the largest BET surface area, which can enhance the adsorptive effect. In the degradation step, the CdSe, CdSe-TiO_2_, and CdSe-C_60_/TiO_2_ composites showed a good degradation effect, as shown in the UV–vis absorption spectra. The CdSe-C_60_/TiO_2_ composites showed good adsorption and degradation effects. A comparison of the decoloration effect of the catalysts showed that the degradation effect can be increased by an increase in the adsorption capacity.

**Figure 9:**
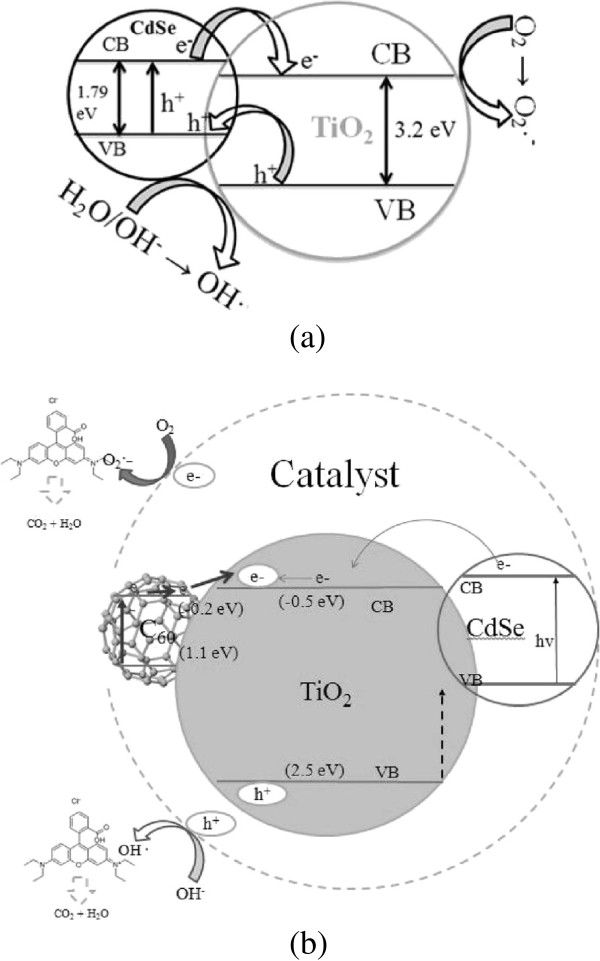
**Schematic diagram of the separation of generated electrons and holes on the interface of compounds.** (**a**) CdSe-TiO_2_ and (**b**) CdSe-C_60_/TiO_2_ compounds under visible-light irradiation.

The enhanced activity is probably attributed to the improved optical absorption and the heterostructure which favors the separation of photo-introduced electron–hole pairs in CdSe-TiO_2_ photocatalyst [28]. Figure 9a shows the scheme of excitation and charge transfer process between CdSe and TiO_2_ under visible-light irradiation. Under irradiation by UV or visible lamp, both CdSe and TiO_2_ can be excited; the generated electrons in CdSe and holes in TiO_2_ are then immigrated to the conduction band (CB) of TiO_2_ and the valence band (VB) of CdSe, respectively. This transfer process is thermodynamically favorable due to the bandgap (both the CB and VB) of CdSe that lie at the upper position than that of TiO_2_. The lifetime of the excited electrons (*e*^−^) and holes (*h*^+^) is prolonged in the transfer process, inducing higher quantum efficiency. Meanwhile, the generated electrons probably react with dissolved oxygen molecules and produce oxygen peroxide radical O_2_^**·**−^, the positively charged hole (*h*^+^) may react with the OH^−^ derived from H_2_O to form the hydroxyl radical OH·. The Rh.B molecule then can be photocatalytically degraded by the oxygen peroxide radical O_2_^·−^ and hydroxyl radical OH · [29,30].

CdSe-C_60_/TiO_2_ composites have the best discoloration effect, which is due to the following reasons: (1) C_60_ is an energy sensitizer that improves the quantum efficiency and increases charge transfer, (2) C_60_ can enhance the adsorption effect during the discoloration processes, and (3) CdSe can provide excited electrons for TiO_2_ and engender hydroxyl radicals (·OH) and superoxide radical anions (·O_2_^−^) with the presence of H_2_O and oxygen. Figure 9b shows a schematic diagram of the separation of photogenerated electrons and holes on the CdSe-C_60_/TiO_2_ interface [31,32].

## Conclusions

Photocatalysts were synthesized successfully using a simple sol–gel method. From the XRD patterns, the cubic crystal structure of CdSe was observed. TEM showed that the surface of TiO_2_ has been coated uniformly with C_60_ and CdSe layers with a C_60_ particle size of approximately 20 nm. The diffuse reflectance spectra indicated that the composites showed strong photoabsorption in the UV–vis range, and the presence of C_60_ enhanced the level of photoabsorption in the visible range. The nitrogen adsorption isotherms show that the added C_60_ can enhance the adsorption effect significantly. The photocatalytic activity of the CdSe-C_60_/TiO_2_ composite was examined by the degradation of MB in aqueous solutions under visible-light irradiation. The CdSe-C_60_/TiO_2_ composites showed good adsorption and degradation effects. Overall, within the limits of the degradation ability, the degradation effect can be enhanced by an increase in adsorption capacity. CdSe-C_60_/TiO_2_ composites also have good photocatalytic activity in cycle experiment which emphasizes the excellent stability of C_60_ and photochemical stability of C_60_-modified photocatalyst.

## Competing interests

The authors declare that they have no competing interests.

## Authors' contributions

ZDM wrote the paper and prepared the samples. LZ, SY, QS, and KU analyzed the sample. KYC performed the TEM. WCO coordinated the study as the corresponding author. All authors read and approved the final manuscript.
